# Copy of a Report on Refrigeration with Rum, in the Cure of the West India Endemic Fever; Addressed to the Commissioners for Transports, &c.

**Published:** 1817-06

**Authors:** Duncan M'Arthur

**Affiliations:** late Physician of the Royal Naval Hospital at Deal, and formerly Surgeon of the Naval and Prison Hospitals at Barbadoes, &c.


*To the Editors of the Medico-Chirurgical Journal & Review.*

GENTLEMEN, Walmer, Deal, 10th May, 1817.

If you shall deem the following Report on Refrigeration with Rum, in the ardent fever of tropical climates, worthy of insertion in your Journal, I beg distinctly to disclaim the most distant intention of detracting from the merits of Dr. Cuming, whose death has been a public loss, and from whose pen, had he lived, the medical world would have received much valuable information.

My intention is solely to shew, by a document written ten years ago, that a mild fever, totally distinct from the yellow fever, prevails in the West Indies, and that we should receive, with some caution, accounts of extraordinary success in the cure of this disease, as the success may depend more on the nature of the fever than on the means employed in the cure.

I am, Gentlemen,
Your most obedient humble servant,
D. M'ARTHUR.

---

*Copy of a Report on Refrigeration with Rum, in the Cure of the West India Endemic Fever; addressed to the Commissioners for Transports, &c. by* DUNCAN M'ARTHUR, *M.D. F.L.S. late Physician of the Royal Naval Hospital at Deal, and formerly Surgeon of the Naval and Prison Hospitals at Barbadoes, &c.*

Naval Hospital, Barbadoes, Nov. 24, 1807.

GENTLEMEN,

Mr. Alexander M'Leay, in his Letter of the 4th of September last, having intimated to me your directions, that I should report to your Honourable Board my opinion of the mode which Dr. Cuming, of the Naval Hospital at Antigua had adopted in the cure of the ardent fever of tropical climates, by refrigerating patients with rum; also enclosing me extracts of Dr. Cuming's letters to your Board of the 10th of May and 5th of June:*

It would afford me sincere satisfaction could I, by my testimony, corroborate the Doctor's report of the success

---

* In 1808, this excellent medical officer lost his wife and two children by the yellow fever; and in a few days after their deaths he fell a victim to the same disease.

which attends refrigerating patients by means of ardent spirits, in the manner he directs; on the contrary, I am disposed to believe, from the following facts, that it is not so beneficial, at any rate not superior to the cold affusion; and when applied at intervals, like the cold affusion, it is attended with most success.

Dr. Cuming, a few months after his arrival at Antigua, made me acquainted with his plan of refrigerating patients with rum; and I was anxious to try its effects on real cases of the endemic of the country, not on the mild fever which occurs at all seasons of the year: but few opportunities offered of trying it early enough to form a judgement of itseffects before I received the directions of your Board. This season we have seldom received patients at the hospital, ill with fever, before the third day, and then a solitary case (probably in the jaws of Death) from some of the ships which arrived in the Bay. The real endemic, on which I had tried its effects, either occurred in patients already in the hospital, or artificers from the dock-yard borne on the Dart's books, and many of these too late to try the effects of the refrigerating plan; however, what experience I had was by no means favourable to the practice.

Lately eighteen cases of fever were sent to this hospital from the Dart; all of them belonged to his Majesty's ship Blonde, and had been some time in the Bay in charge of a prize. These men were only nine months in the country, and were sent on shore from twenty-four to forty-eight hours after the attack of fever, and were all equally ill on their admission to the hospital.

Eleven of these, taken indiscriminately, whose skin was hot and dry, eyes red, excessive head-ach, pain very severe of the loins, shooting forward to the umbilicus, had the common cold affusion applied in the usual manner; five, under similar circumstances, were refrigerated for six hours with rum, and on the following days, when heat of skin indicated the propriety, it was repeated. Two cases, who perspired freely from the beginning, had neither the cold affusion nor refrigeration with rum; all of them, in every other respect, were treated alike, as symptoms pointed out. Of the first eleven, two died; of the next five, two died, and one was a long time doubtful; one of the remaining two, who were not refrigerated with rum nor by the cold affusion, died contrary to every expectation.

From several comparative trials (which I have since made and am now making), I am warranted to conclude, that it is not equal to the cold affusion, when judiciously applied every second hour, and the quantum of shock is considerable by the water being dashed on the patient from a height; and that it is most useful when employed like the cold affusion, that is to say, to suffer time for the vascular system to re-act after each application.

The following effects, during the continuance of the cold, have been observed: When the spirit is first applied, there is a very considerable degree of cold, but upon the whole agreeable to the patient; a longer continuance, and the sensation of cold becomes very unpleasant, and at last is almost insupportable, inducing shivering almost amounting to the horror in the cold stage of an intermittent. In some instances it has reduced the pulse in frequency, but not much; in others, it has increased it, and in one case caused it to intermit every fifth or sixth stroke, but invariably, from the pulse being full and expanded, it induced a contracted and rigid pulse; but in no case has the patient been relieved, either by the refrigeration with rum or the cold affusion, before there has been several free and copious evacuations from the bowels, and a gentle perspiration has broke out.

I have never been able to use the refrigeration with rum before the first six hours of the fever, or until it was completely formed, nor do I think it can possibly be used much earlier in common hospital practice; but if the surgeons of the navy have so powerful an antidote to ardent fever in the rum bottle, as Dr. Cuming so sanguinely expects, there cannot be any necessity for recommending patients to be sent early to an hospital, because the refrigeration can be employed equally well on board a ship, as on shore; and in this way the discovery would not only be a benefit to mankind, but a great saving to government. Should I ever have reason to alter my opinion of the new refrigerating plan, I shall have much pleasure in communicating the same to your honourable Board. It must appear strange, that the same practice pursued in two hospitals so contiguous as Antigua and Barbadoes should differ so widely in its results; I feel it my duty to explain the reason of this difference.

From the mortality which has attended fevers in this country, every stranger, whether a medical man or not, alarmed for his own or his friends safety, considers every ephemeral febrile complaint which they meet on their first arrival as the fatal fever of the West Indies; it is therefore proper to state to your Board, that although we meet with examples of this fever at all seasons of the year in strangers, exposed to some strong predisposing or exciting cause, yet these are solitary instances, and the fever is never general but between the middle of July and the end of December. During this period, the natives and seasoned Europeans are not exempted from it, although some of them die with every symptom of the yellow fever, yet its effects are mild compared to the havoc it makes in constitutions unassimilated to the climate. A ship of war, or a regiment of soldiers, arriving from Europe about the beginning of January, are exempt from the fever till about the middle of July, and sometimes two or three months later. I am therefore of opinion, that at the time Dr. Cuming made his communications of the 10th of May and 5th of June, he had met with few cases of the endemic of the country, and that the fevers which were previous to that period under his care were the mild febrile complaints which occur at all seasons of the year.

That many of these fevers are sent to the hospital at Antigua, I believe from common report. The capstan-house, where ships' companies are lodged while ships are repairing, is so uncomfortable that few captains will suffer any of their men (who would otherwise be ordinary objects for the sick list) to remain there, where a complaint, which might be mild at first, would, from want of accommodation and air, become a fatal one; they are therefore sent to the hospital. Dr. Cuming having so lately arrived in the country, may have mistaken the mild for the fatal fever of the West Indies; indeed, I am assured the bad fever never appeared at Antigua before the month of June: of this fact you may be certified, by a reference to the quarterly books of that hospital for a series of years past.*

Even during the unhealthy season, if the ships' companies are assimilated to the climate, although many fevers occur, the mortality is trifling compared to the mortality on board ships lately arrived from Europe. The following fact will prove how mild the fevers of this country are at other seasons of the year:

In March or April last, H.M.S. Northumberland, lying in this bay, bearing the flag of Rear Admiral the Ho

---

* Recent occurrences at Antigua lead me to think the sickly season there varies. June 6th, 1808.

nourable Sir Alexander Cochrane, had a number of men suddenly attacked with fever, which caused some alarm; I went on board with Dr. Dickson, examined the sick, and we were both of opinion, that as the island was healthy, the season for the appearance of the endemic fever had not arrived; also, most of the patients having been ill formerly with the fatal fever of the country, that no danger was to be apprehended. The result proved we were right: in a few days, there were between 120 and 130 in the sick list; not a man was sent to the hospital; and they all recovered under the judicious practice and humane care of Dr. Dickson.

Many a surgeon, unacquainted with the diseases of this country, would have sent the sick to the hospital; or, if he kept them on board, might have published to the world his success in the cure of the yellow fever.

I have already stated, and now repeat, that I have not found the continued refrigeration with rum so good an auxiliary in the cure of fever as the cold affusion; but it is more easy in its application, and if employed at intervals, like the cold affusion, may be of use where water cannot conveniently be procured.

I gave every publicity in this island to Dr. Cumings's mode of curing fever by refrigeration with rum, and every opinion which I have heard of its utility is in coincidence with that which I have expressed.

The allaying of vomiting by means of aq. menthæ, opium, blistering, and the effervescing saline draughts, are not new, and therefore require no opinion from me respecting their use; I have only to lament that they have not been so successful in my hands, and in those of others in allaying vomiting in the real yellow fever as they appear to be in Dr. Cumings's.

I have the honour to be, with every respect,

Gentlemen,

Your most obedient and very humble servant,

(Signed) DUNCAN M'ARTHUR.

Commissioners for Transports, &c. &c. &c.

